# An efficient protocol to use feedback-controlling digital microfluidic fluorimetric sensor for detection of mercury (II) in coastal seawaters

**DOI:** 10.1016/j.mex.2019.06.006

**Published:** 2019-06-14

**Authors:** Shuang Han, Xianming Liu, Lin Wang, Yunhua Wang, Guoxia Zheng

**Affiliations:** a Chemical and Environmental Engineering Institute, Dalian University, Dalian, 116622, China; b Dalian Institute of Chemical Physics, Chinese Academy of Sciences, Dalian, 116600, China; c Medical School, Dalian University, Dalian, 116622, China; d Environmental Micro Total Analysis Lab, Dalian University, Dalian, 116622, China

**Keywords:** Digital microfluidics, Feedback-controlling, Fluorimetric sensor, Hg detection, Seawater

## Abstract

Mercury ion is a highly toxic anthropogenic pollutants and has serious well-known effects on human. There is an ever-growing demand for convenient detection of mercury driven contaminants in environment, including coastal seawater. However, most of the reported methods are instrument-based and are not easy for portable detection. Our protocol described an efficient Digital Microfluidics method for detecting mercury in coastal seawater samples. It combined the miniaturization/automation potential of digital microfluidics and the sensitivity of fluorescence probe. To overcome a potential risk of driven failure, induced by diversity ion ingredients in seawater, a feedback control loop was included into control system. The method showed satisfied stability and selectivity in Hg sensing under high salinity condition, with the sensitivity of Hg^2+^ at the parts-per-billion level and total testing time of less than 20 s. With the advantages of being fast, amenable to automation and low cost, this protocol is promising for the formation of simple and rapid sensor device, especially for a routine monitoring and emergency detection of Hg/or other metals in coastal waters.

## Graphical abstract

**Figure:**
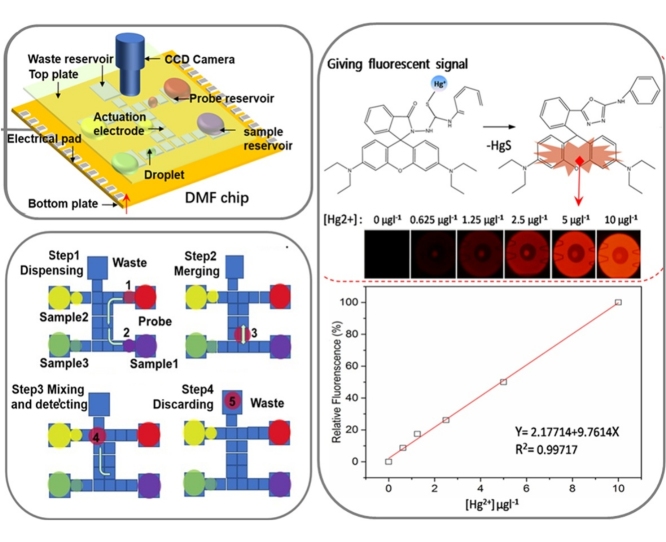

**Specifications Table**

**Table:** 

Subject Area:	*Environmental Science*
More specific subject area:	*Environmental Micro Total Analysis*
Protocol name:	Fluorimetric microdetection of mercury with feedback- controlling DMF
Reagents/tools:	All chemicals used in synthesis experiment were of analytical grade. The deionized water (MQ) was prepared from Milipore1 (Merck KGaA Darmstadt, Germany). The known rhodamine B hydrazide was prepared according to a literature procedure [2].The metal ion solution was prepared from its perchlorate salt. DMF chip was fabricated using a bottom plate of Cr-coated glass and a top plate of ITO glass. The SU8-3005 was coated on Cr-glass as a dielectric layer. Teflon-AF was spin-coated on the surfaces of the ITO glass and SU8-3005, respectively, as a hydrophobic layer. The two plates are separated by an ITO coated PEF film.
Experimental design:	The DMF chip is mounted on the chip holder. Three samples without extra complex pretreatment and mercury fluorescence probe (RS) were loaded onto reservoir electrodes. The control system generates the droplet routing plan and actuates the droplet using the EWOD force in real time. A 1μl RS droplet and a 1μl sample droplet were dispensed from the reservoir electrodes, respectively. These two droplets were merged and actively mixing by reciprocating motion between adjacent electrodes to implement fluorescent color reaction selectively and rapidly. The mixing droplet was transferred to detecting position and the fluorescent image was captured for fluorescence intensity analysis. The droplet then was transferred to the waste reservoir electrode and discarded.
Trial registration:	NA
Ethics:	No ethical issues involve

Value of the Protocol

• An automatic method for Hg/or other metals analyses in coastal seawaters is proposed.
• The method is very quick and easy, it simplifies the analyzing process into droplet actuation (i.e. dispensing, merging, and active mixing).
• A feed-back loop was included in the control system to insure the actuation of seawater droplet.
• The method gives fluorimetric reaction that can be useful for the selective and qualitative assessment of Hg contamination in high salinity setting.

## Description of protocol

**Fig. 1:**
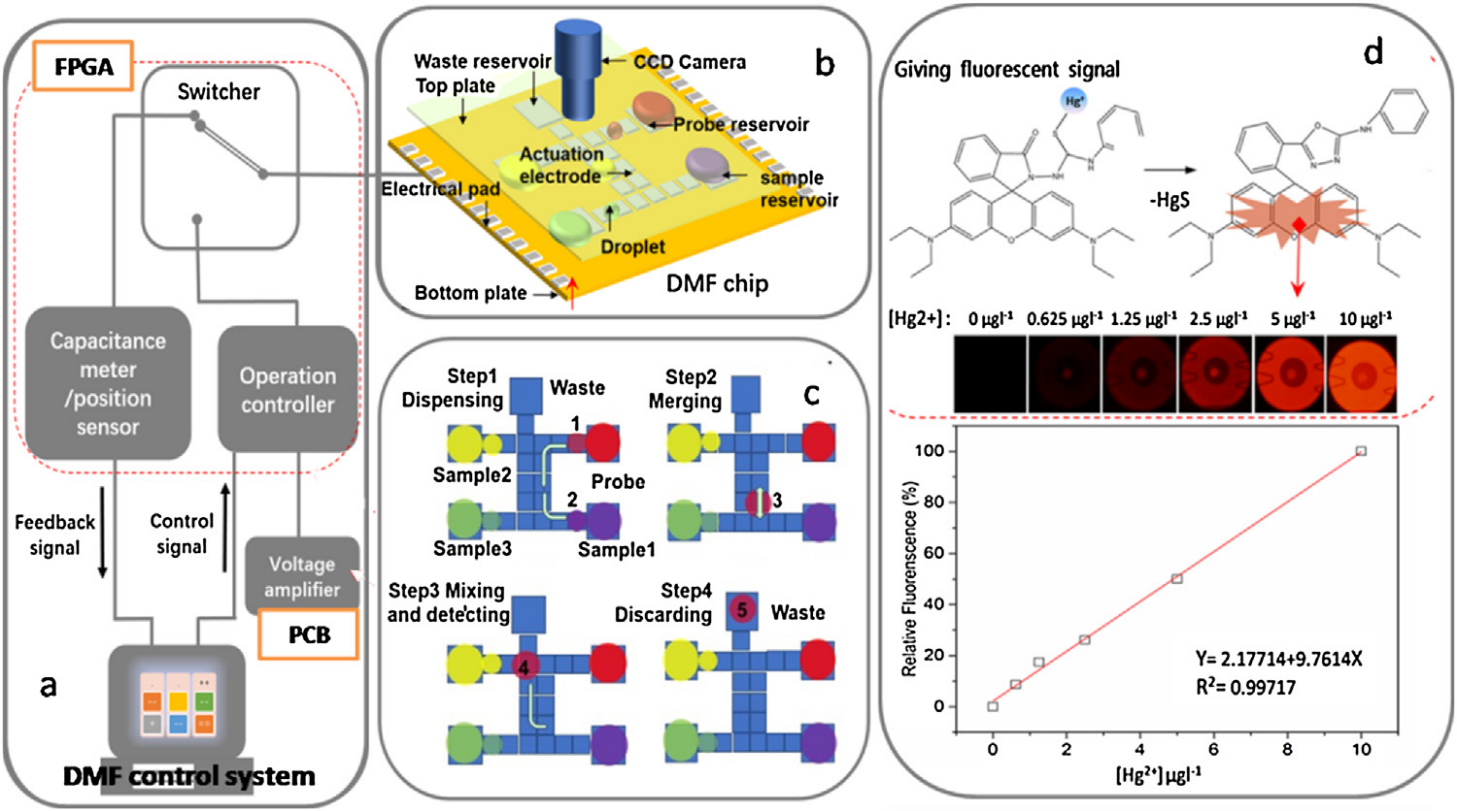
The mercury assay process based on DMF sensor through precisely controlling over seawater droplets (i.e., dispensing, merging, and active mixing) to implement fluorescent color reaction selectively and stoichiometrically. The DMF sensor device including two main parts of (a) DMF control system and (b) DMF chip. (c)Test procedure on chip (d) Detecting of Hg-RS mixture.

The schematic representation of the process was shown in Fig. 1. To measure the Hg concentration on DMF sensor (Fig. 1 a), the standard solution or samples and mercury fluorescence probe (prepared in CH_3_CN) were loaded onto reservoir electrodes. Three samples without extra complex pretreatment could be loaded simultaneously (Fig. 1b). The analyzing process was automatically performed by the droplet actuation which was simplified into dispensing, merging, and active mixing (Fig. 1c). A droplet, working as a micro mixer, can significantly improve mass transfer, shorten the reaction time and improve the yield of the product. Then Hg-RS mixture was transferred to detection position. Images were captured by fluorescence microscopy with a CCD camera and was converted to eight-bit grayscale mode by Image-Pro Plus (V6.0 for Windows XP, Media Cybernetics, Inc., USA) (Fig. 1d). The total detection time was less than 20 s. The detailed test procedure is described as followed (Fig. 1c). The video of analyzing process was available in ESI video.

Step1: Samples and the RS probe were loaded onto the corresponding reservoir electrodes. A 1 μl RS droplet and a 1 μl sample droplet were dispensed from the reservoir electrodes respectively.

Step2: RS droplet and sample droplet were merged and actively mixed by reciprocating motion between adjacent electrodes.

Step3: The mixed droplet was transferred to detecting position and the fluorescent image was captured for fluorescence intensity analysis.

Step4: The droplet then was transferred to the waste reservoir electrode and was discarded.

Step5: Additional step of pretreatment (evaporation-concentration) will be involved if the mercury concentration was beyond the sensitivity.

The operation principle of the DMF sensor was described as follows:

Step1: The DMF chip with droplets was mounted on the chip holder.

Step2: Each droplet was identified and located.

Step3: The control system generated the droplet routing plan and actuated the droplet using the EWOD force in real time.

The control system was based on a real-time feedback positioning mechanism (Fig. 1 a). The embedded protocol can precisely track the static or dynamic position of each droplet, being capable to drive complex droplet transportation. The developed DMF chip consisted of two parallel plates (63 × 33 mm). 24 electrodes were patterned on the bottom plate. (Fig. 1b), These electrodes were driven by control electronics on a PCB board (PCB, Fig. 1a), switching between the voltage-actuation mode and the droplet-sensing mode. For the latter, the field programmable gate array (FPGA, Fig. 1a) scans the capacitance of each electrode by using an oscillator outputting variable-frequency pulses. The collected frequency information is transferred to the operation unit (i.e., the computer) for real-time computation, and finally feedback to the DMF chip.

## DMF chip fabrication

**Fig. 2:**
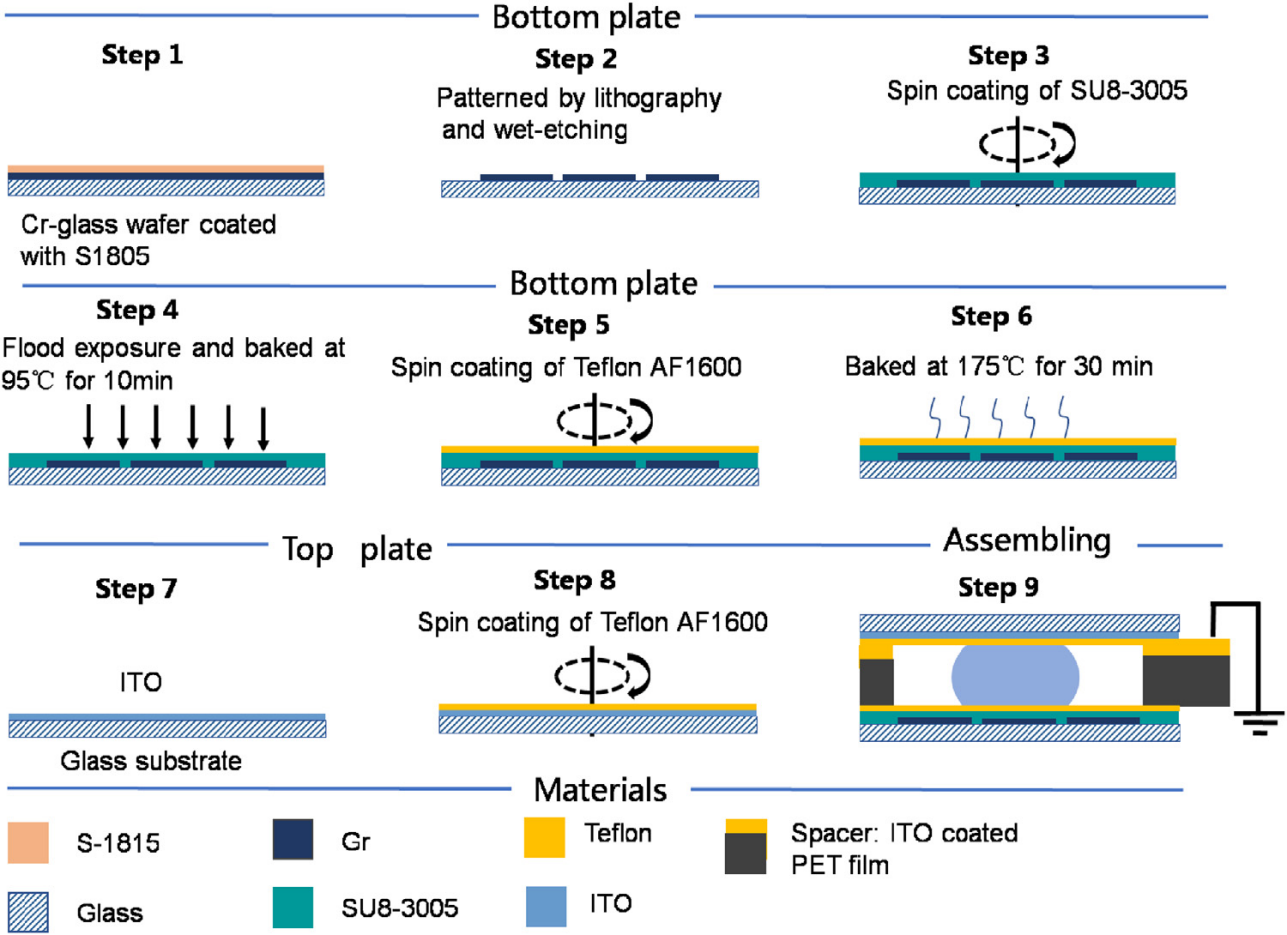
Fabrication procedure of two-plate DMF chip.

The fabrication procedure was shown in Fig. 2. Step1-2: The electrodes on the bottom plate were drawn in AutoCAD (Autodesk, Inc.) and patterned onto Cr-coated glass with standard lithographical plus wet-etch methods. Step3: The SU8-3005 dielectric layer covering electrodes was obtained by spin coating (1500rmp, 60 s). Step4: The substrate was exposed to UV for 20 s. After post-baked on hot plate (95 °C, 10 min), the substrate was subsequently placed in an oven (180 °C, 2 h) for curing. Step5-6: Teflon-AF (2% wt/wt in Fluorinert FC-40) was spin-coated (1500 rpm, 30 s) onto the substrate followed by baking on a hot-plate (175 °C, 30 min). Step7-8: Using the same experimental protocol, Teflon-AF was spin-coated onto the surface of the ITO glass(top plate of the chip). Step9: The top and bottom plates were assembled together to form a DMF chip. The two plates were separated by a ITO coated PET film. The thickness of the film determined the distance between the two plates. The ITO film was connected to ground of power supplier.

## Hardware of DMF control system

The feedback DMF sensor consists of two main parts, i.e. DMF chip and DMF control system (Fig. 1 a and b).

The DMF chip consisted of two parallel plates (63 × 33 mm). The top layer of the DMF chip was an ITO glass, featuring high electrical conductivity on the bottom side. ITO coated PET film was used as spacer of top and bottom plate, with the ITO layer on up-side of the film and was connected to ground of power supplier. The bottom layer was an glass layer, where 24 electrodes were patterned on the upper surface.

**Fig. 3:**
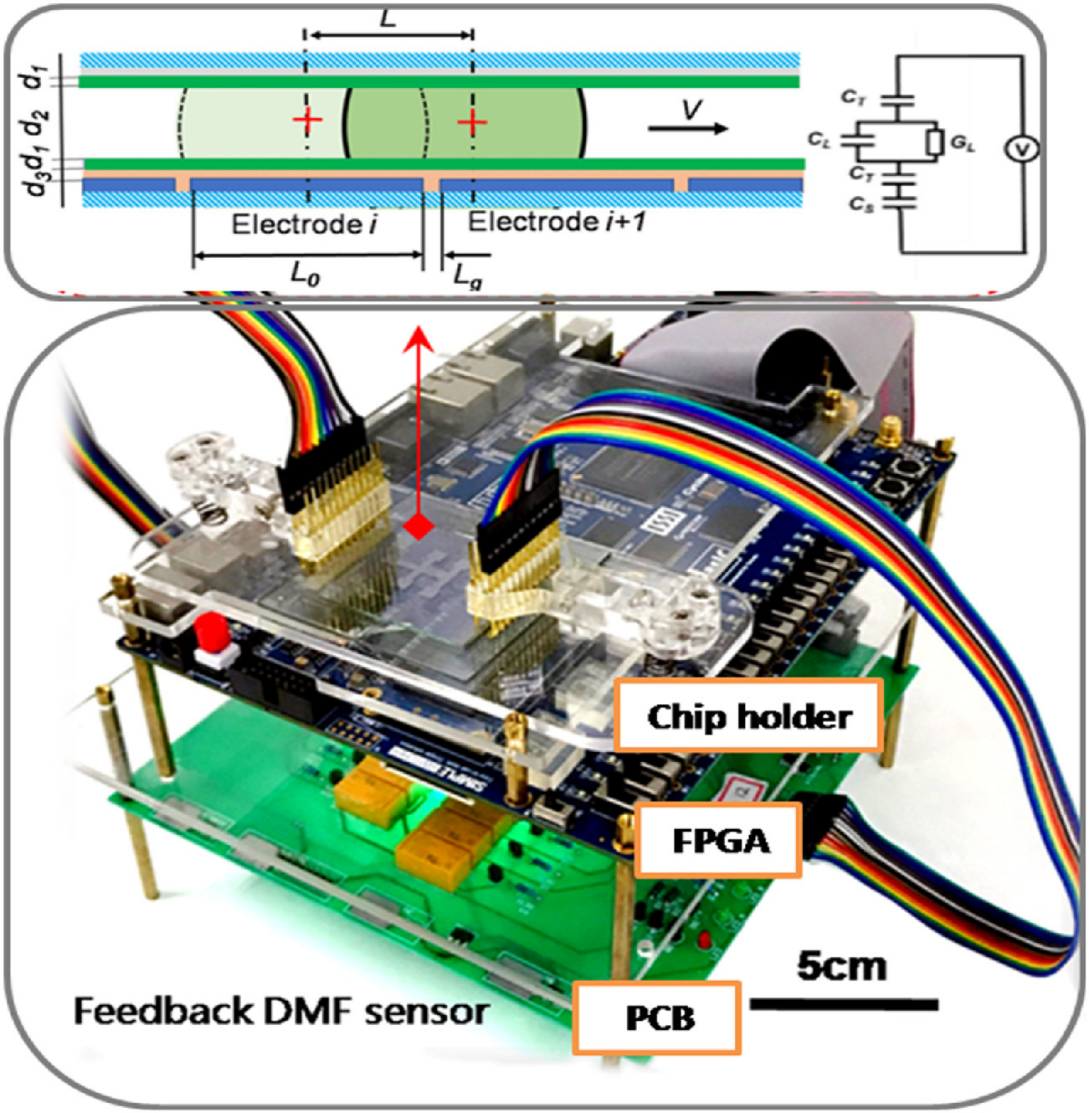
The hardware of the DMFcontrol system included three operation layers: DMF chip holder (top layer), field-programmable gate array (FPGA) board (FPGA, mid layer) and control electronics board (PCB, bottom layer) and The DMF chip holder was to mount the DMF chip. The control electronics on the printed circuit board (PCB) was used to apply driving voltages (acquire capacitance information) to (from) the DMF chip. The FPGA acquired the signal from the control electronics and transferred it to the computer. The IC testing clips connectwed the DMF chip to the control electronics. The relay array and multiplexers switched the DMF chip between the driving voltages (actuation) and oscillator (sensing).

The DMF system consists of three operation layers: DMF chip holder (top layer), FPGA board (mid layer) and control electronics board (The printed circuit board, PCB, bottom layer) (Fig. 3). (1) Chip Holder. The main body (Fig. 3, top layer) is a PMMA (Acrylic) plate and is ergonomically sized as 15 × 7 × 2.5 cm (L × W × H) to be fitted for the human operator. The DMF chip is fixed by two 12-pin IC Testing Clips for droplet sensing and voltage actuation. (2) FPGA Board. The hardware control (Fig. 3, mid layer) was implemented by a FPGA board (Altera Cyclone® II 2C70 device). The hardware language was Verilog. The internal units included data transfer, frequency detection, data collection and operation controller, which were capable to perform: droplet transportation sequence programming, feedback-based position correcting, and controlling of switching between actuation mode (operation controller) and position sensor mode, integrating the data and output actuation signal accordingly. (3) Control Electronics (PCB). (Fig.3, bottom layer) was drawn with Altium Designer with a dimension of 20 × 20 cm (L × W). The control electronics was used to interface the DMF chip with the FPGA. There were 25 electrically-controlled relays mounted on the PCB for connecting the DMF chip with the driving voltage or the oscillator.

## Feedback-control for droplet-position correction

The improvement of droplet movement fidelity was evaluated by the moving completion to the target electrode. Actuation with and without feedback controlling were tested on various droplets of DI water, NaCl solution, real seawater samples and condensed seawater. To monitor the motion of droplet, the capacitance dependent position feedback method was performed as previously described [1].

Briefly, the capacitance of each electrode was scanned after each actuation. The capacitance of two adjacent electrodes was marked as *C_i_* and *C_i+1_* (as shown in Fig. 3). The position of droplet could be estimated by equation:

(1) $$L≅\frac{C_{i+1}}{C_{i}+C_{i+1}}\left(L_{0}+\right)Lg)$$

Where *L* was the dynamic position of the droplet from the start point. $L_{0}$ was the length of the electrode and *Lg* was the gap between two adjacent electrodes. Since the *L* (≥ 1 mm) was much larger than *Lg*(≤ 50 μ m), Eq. (1) could be simplified as:

(2) $$L≅\frac{C_{i+1}}{C_{i}+C_{i+1}}L_{0}$$

When L = $L_{0}$, $\text{X}=\frac{L}{L_{0}}=\frac{C_{i+1}}{C_{i}+C_{i+1}}$ = 100%, we considered that the droplet arrived the destination.

Where X was displacement percentages calculated as leading edges of the droplet with respect to their previous positions. The droplet velocity (*V*) was then given by:

(3) $$V=\frac{L_{0}X}{t}$$

Where *t* is charging time.

## Synthesis and characterization of fluorescent probe

**Scheme 1:**
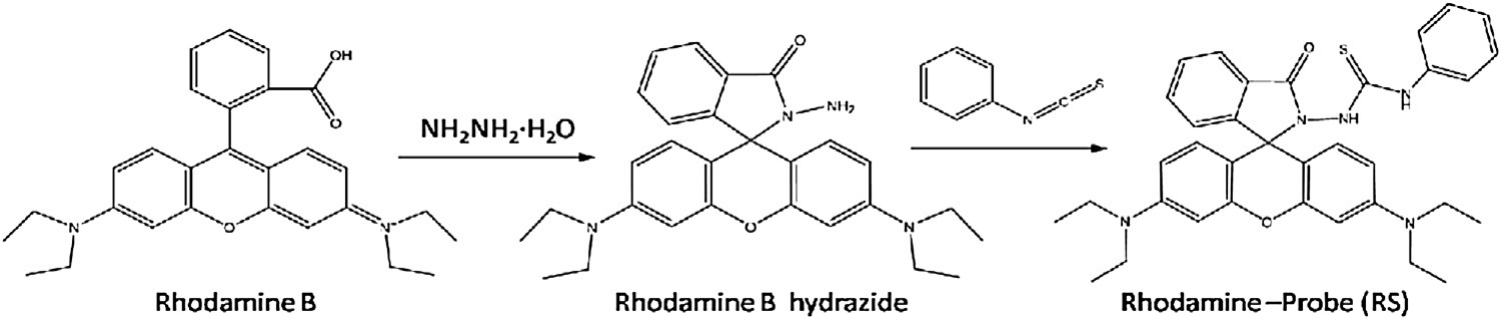
The reaction process of the RS.

**Fig. 4:**
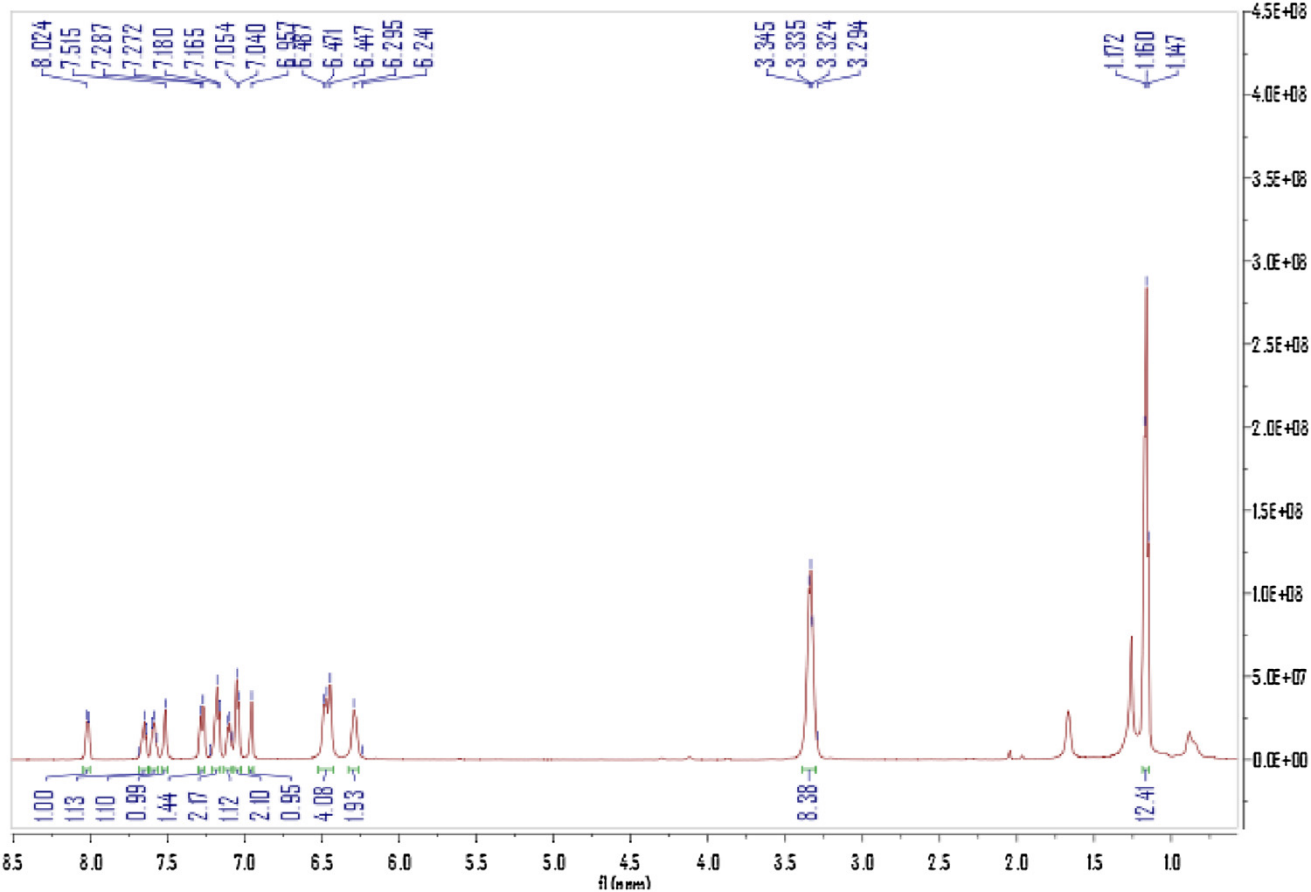
^1^H NMR of theRS in DCl_3_.^1^HNMR(CDCl3, 500 MHz): *δ*1.160(t, 12H, NCH_2_CH_3_), 3.335(m, 8H, NCH_2_CH_3_), 6.295(d, 2H, xanthene H), 6.447--6.487(t, 4H, xanthene H), 6.957(S, 1H, NH), 7.047(d, 2H, ArH), 7.097(t, 1H, ArH), 7.172(t, 2H, ArH), 7.279(d, 1H, ArH), 7.515(S, 1H, NH), 7.585(t, 1H, ArH), 7.653(t, 1H, ArH), 8.017(d, 1H, ArH).

**Fig. 5:**
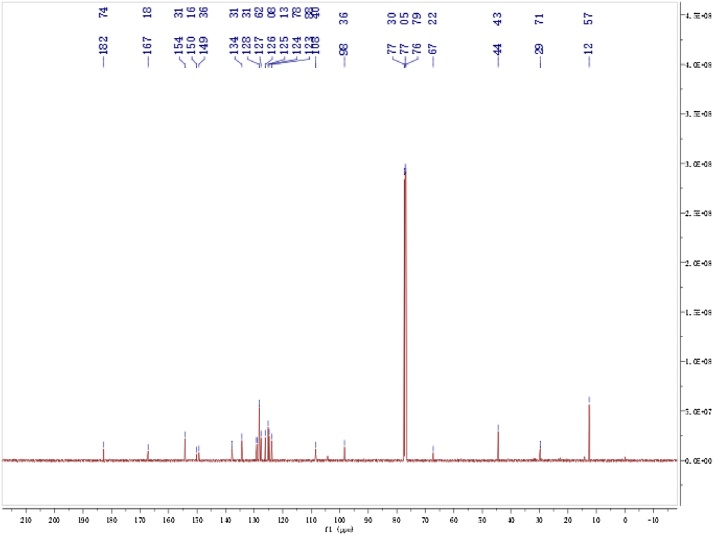
^13^CNMR of the RS in DCl_3_.^13^CNMR(CDCl_3_, 300 MHz) *δ*12.565, 44.434, 67.217, 98.355, 108.896, 124.780, 125.133, 126.076, 127.621, 128.309, 134.312, 149.362, 150.159, 154.311, 167.185, 182.745. ESI-MS:*m/z*: 592.1(M^+^).

The Rhodamine B hydrazide (200 mg, 0.44 mmol) in Dimethylformamide (1.5 ml) was added to a solution of phenyl isothiocyanate (0.1 ml, 0.65 mmol) in Dimethyl- formamide (1.5 ml). The reaction mixture was stirred for 6 h at room temperature (Scheme 1) [2]. After the solvent was evaporated under reduced pressure, the crude product was column chromatographed on silica-gel (elution with hexanes/ EtOAc/CH_2_Cl_2_ = 4:1:1) to give the compound of 1- Rhodamine Bhydrazide-3 -phenylthiourea (RS) as a white solid (187.6 mg, isolated yield: 72%). NMR spectra were recorded on a Bruker DRX-400 spectrometer using TMS as an internal standard. ^1^HNMR and ^13^CNMR of the RS were showed in Fig. 4 and 5 .

**Scheme 2:**
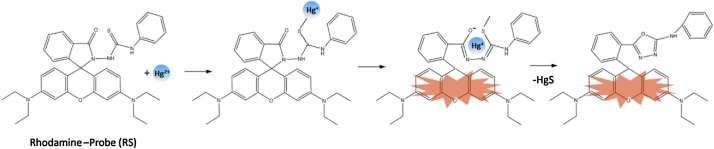
Sensing mechanism for the fluorescent changes of the probe RS to the Hg^2+^. Hg^2+^ can be selectively bind to RS, leading to the spirolactam ring “close-open” transformation and generation of the xanthene form and fluorescence.

**Fig. 6:**
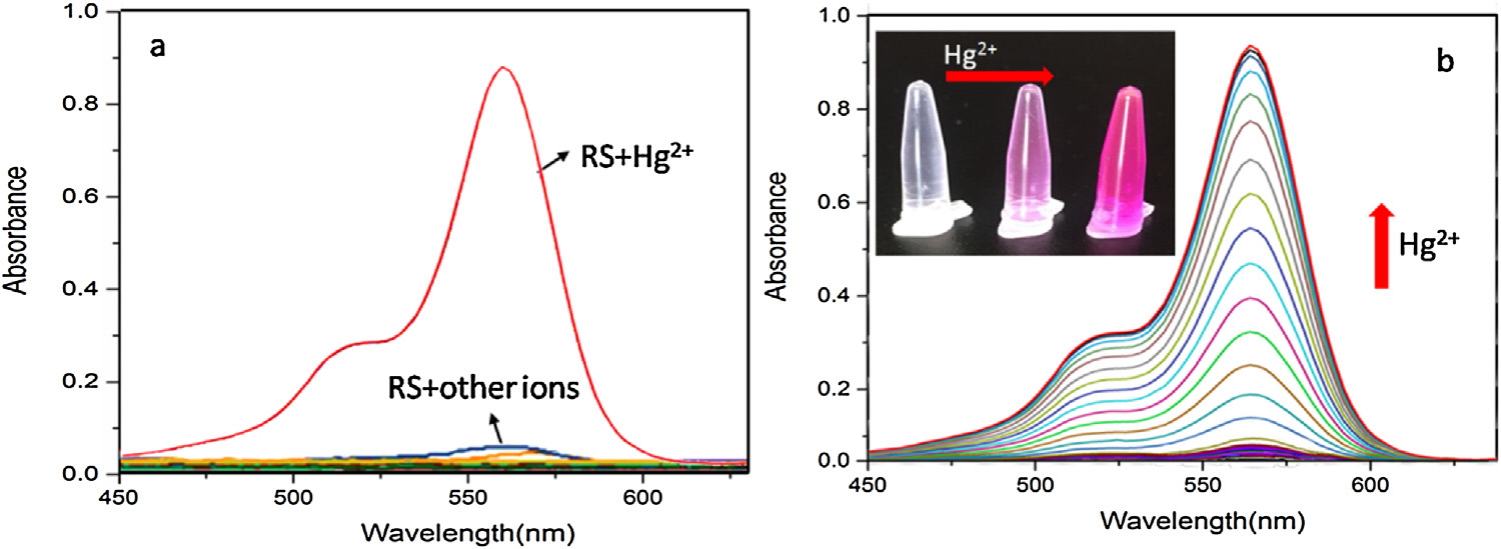
(a) UV–vis absorption spectra of the receptor RS (10 μM) observed upon the addition of 100 μM ions (Na^+^, Ag^+^, Ca^2+^,Mg^2+^, Cu^2+^, Zn^2+^, Cd^2+^, Ni^2+^, Mn^2+^,Fe^2+^, Cr^6+^, Fe^3+^, Pb^2+^and ClO_4_^−^) in CH_3_CN/H_2_O (5 :5, v/v). (b) UV–vis titration spectra of RS (10 μM) upon incremental addition of0 to 100 μM Hg^2+^ in CH_3_CN/H_2_O (5: 5, v/v). Inset picture: the color changes of RS to Hg^2+^.

Based on the irreversible desulfurization of thiocarbonyl promoted by mercury ions, the synthesized RS detected Hg^2+^ with high selectivity and high sensitivity. A likely sensing mechanism based on the Hg^2+^ triggered spiro ring-opening process was proposed in Schemes 2. The UV–vis absorption experiment were conducted in CH_3_CN/H_2_O (5:5, v/v), using Spectra max plus 384 (Molecular Devices, CA, US). UV–vis spectra were recorded for RS in the presence of various ions including Hg^2+^. A significant change in the UV–vis absorption spectrum pattern at 562 nm in the presence of Hg^2+^ among all the tested ions including Ag^+^, Ca^2+^, Mg^2+^, Cu^2+^, Zn^2+^, Cd^2+^, Ni^2+^, Mn^2+^, Fe^2+^, Cr^6+^, Fe^3+^, Pb^2+^ and ClO_4_^−^, indicating a high selectivity of RS toward Hg^2+^ (Fig. 6a). The UV–vis absorption peak around 562 nm increased with increasing the Hg^2+^concentration from 0 to 100 μM (Fig. 6b). With the molecular ring opening trigged by Hg^2+^, the solution color change can be observed by the naked eye and turned from colorless to pink (the inset picture of Fig. 6b)

## DMF actuation of sea water droplet

DMF showed advantages in reconfigurability, scalability, mechanical simplicity and low energy consumption, holding particular promise for portable systems. In an ideal DMF system, application of potential would drive droplet to move onto energized electrode. However, in real systems, droplets were sometimes observed to resist movement onto particular electrodes. Actuating was difficult to be precisely controlled, as it was affected not only by operation parameters (e.g., actuation frequency, driving voltage, and charging time) but also by many random parameters (e.g., droplet content, surface roughness of hydrophobic coating, dielectric layer properties and thickness, as well as their long-term time dependency) [1,3]. Each one of these factors changed the droplet response. Seawater droplet, the detecting target in this research, containing various chemical compositions, was a special and complex system with feature of high salinity. Due to diversity of chemical ingredients involved, which may be various in viscosity, conductivity and /or permittivity, necessary actuation may be various accordingly. To overcome a potential risk of driven failure, which may be induced by diversity contents in seawater, a feedback control loop was included into DMF control system. All seawater droplets were addressed by the droplet-derived real-time capacitance scanning. When a movement failure was observed, additional charging time will be applied repeatedly, until the droplet reach the destination. As listed in Table 1, the results confirmed that droplet movement fidelity is dramatically improved for NaCl solutions (e.g. 92% completion without feedback and 100% completion with feedback) and seawaters (e.g. 87% completion without feedback and 99% completion with feedback) as well as condensing ones (e.g. 91% completion without feedback and 99% completion with feedback).

**Table 1:** Transport of seawater with and without feedback control.

	Maximum % completion	
	No feedback	With feedback
DI water	91%	99%
NaCl solution (35‰)	95%	100%
NaCl solution (20%)	92%	100%
Sample 1 with 32.14‰ salinity	87%	100%
Sample 2 with 32.10‰ salinity	90%	100%
Sample 3 with 32.06‰ salinity	93%	99%
Sample 1 (×5 condensing)	91%	99%
Sample 1 (×10 condensing)	90%	100%

## Fluorescence response of RS to Hg in high salinity setting

**Fig. 7:**
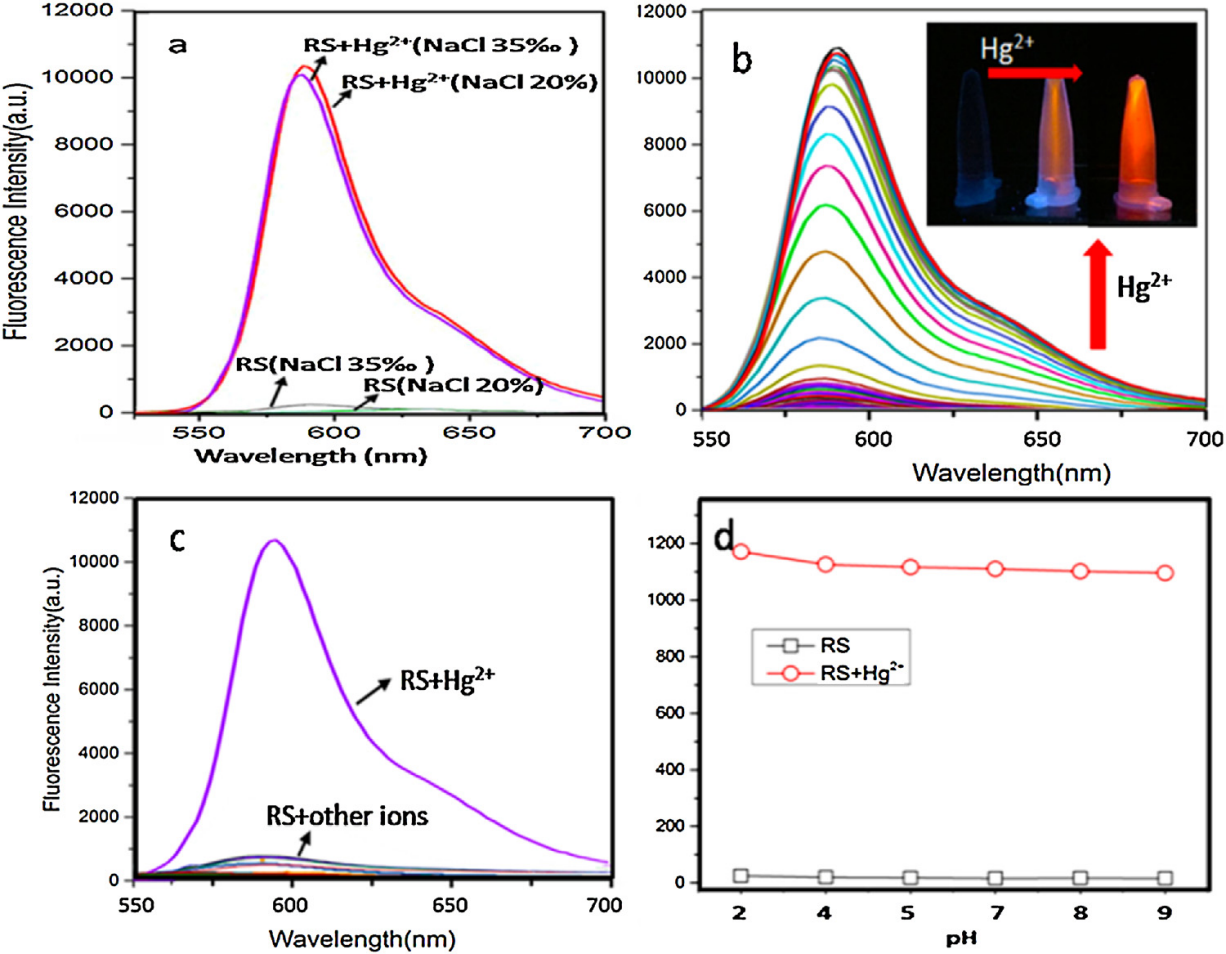
Working suitability measurement of RS to Hg detection in high salinity setting and various pH. (a) Effect of high salinity on the emission intensity of free probe and [Hg^2+^-probe] system. (b) Fluorescence titration spectra of RS (10 μM) with the addition of 0–50 μM of Hg^2+^. Inset picture showed the visual fluorescence color changes of RS to Hg. (c) Changes of the fluorescence emission of the probe RS (10 μM) observed upon the addition of ions (Na^+^, Ag^+^, Ca^2+^,Mg^2+^, Cu^2+^, Zn^2+^, Cd^2+^, Ni^2+^, Mn^2+^,Fe^2+^, Cr^6+^, Fe^3+^, Pb^2+^ andClO_4_^−^) (100 μM). (d) Effect of pH on the emission intensity of free RS and [Hg^2+^-RS] system.

Beside the DMF technology, the fluorescent probe is another important part of the detection sensor platform. To study the stability and selectivity of the probe in high salinity setting, the fluorescence spectra of the probe response toward Hg^2+^ in NaCl solutions (35‰ and 20%) was evaluated (Fig. 7). Excitation of probe in CH_3_CN/NaCl solution (5: 5, v/v, concentration of 35‰ and 20%, respectively) did not show any significant fluorescence emission over the range from 550 to 700 nm (Fig. 7a). This supported the fact that in the absence of Hg^2+^, the probe receptor remained in the ring closing form under high salinity condition. A significant fluorescent response was observed in the presence of Hg^2+^ under high salinity condition (CH_3_CN/NaCl solution, 5:5, v/v, salinity of 35‰ and 20%, respectively). There was no any noticeable difference between them (Fig. 7a). Results clearly showed that RS worked stably under a high salinity condition. The titration was performed with increasing concentration of Hg^2+^. As described in Fig. 7b, the fluorescence intensity has enhanced about 130-fold with the addition of 50 μM Hg^2+^ ions, showing the high sensitivity and stoichiometry of RS toward Hg^2+^ in high salinity setting (35‰ solution) (Fig. 7b).

The fluorescence spectrum was also recorded of 10 μM RS in presence of Hg^2+^ (100 μM) and other ions (100 μM) under high salinity condition (Fig. 7c). The binding induced ring opening of RS and generation of a xanthenes moiety that was highly selective toward Hg^2+^, hence a significant fluorescent response was observed in the presence of Hg^2+^. There was no any noticeable spectral changes for other tested ions (Ag^+^, Ca^2+^, Mg^2+^, Cu^2+^, Zn^2+^, Cd^2+^, Ni^2+^, Mn^2+^, Fe^2+^, Cr^6+^, Fe^3+^, Pb^2+^ and ClO_4_^−^), confirming the high selectivity of RS to Hg^2+^ in high salinity solution (Fig. 7c).These results clearly showed that this fluorescent probe has worked stably, sensitively and selectively under a high salinity condition, which demonstrated the suitability of this RS for Hg^2+^ detection in seawater system (average salinity 35‰). The fluorescence spectra of the probe responses toward Hg^2+^ under different pH conditions were also evaluated (Fig. 7d). It showed that this probe kept stable in a broad range of pH 2.0–9.0.

## On-chip Hg detection

On-chip Hg^2+^ detection experiments were carried out to verify the suitability of the whole DMF sensor to the Hg^2+^ detection in seawater. For comparing, atomic fluorescence method was used for Hg regular detection. As described in Fig. 1, the fluorescence intensities were enhanced with the addition of Hg^2+^ ions. The fluorescence intensities obtained by five separate measurements were plotted against the Hg^2+^ concentration to generate a standard curve (Fig. 1d). The fluorescence titration profile of RS (0.1 μM) with Hg^2+^ demonstrates that detection of Hg^2+^ is at the parts per billion level, which suggest that this method can serve as the foundation of practical chemo dosimeters for determining Hg^2+^ concentrations in coastal environments. Under these conditions, the fluorescence intensity of the RS was nearly proportional to the amount of Hg^2+^ added. Line regression analysis was applied with *R*^2^ of 0.997 (Fig. 1d). The limit of detection was calculated as 3.5 × 10^−3^μM from the 3σ method (the limit of detection = 3σ /slope). Considering the robustness of our DMF sensor (liberated from tubing system and ability of precisely fluidic controlling and giving fluorescent signal in high salinity setting), a minimal pretreatment will be involved if the mercury concentration was beyond the sensitivity. Evaporation-concentration was the only step rather than complex pretreatment used in traditional analysis for seawater quality with satisfactory result and low detection limit [[4], [5], [6], [7], [8]].

Mercury was automatically detected by our DMF device. The minimization of controller, the imaging system, signal generator and amplifier will produce a portable device. By further integration with machine vision, which could capture and analysis the images automatically, a sample-to-results solution will be achieved.
